# Antigingivitis, Desensitizing, and Antiplaque Effects of Alkaline Toothpastes: A Randomized Clinical Trial

**DOI:** 10.3390/dj11040096

**Published:** 2023-04-04

**Authors:** Nina Novozhilova, Elena Andreeva, Maria Polyakova, Irina Makeeva, Inna Sokhova, Vladlena Doroshina, Alexandr Zaytsev, Ksenia Babina

**Affiliations:** 1 Department of Therapeutic Dentistry, I.M. Sechenov First Moscow State Medical University (Sechenov University), 119991 Moscow, Russia; 2 Institute of Linguistics and Intercultural Communication, I.M. Sechenov First Moscow State Medical University (Sechenov University), 119991 Moscow, Russia

**Keywords:** toothpastes, dentin sensitivity, fluorides, gingivitis, hydrogen-ion concentration

## Abstract

Gingivitis is a widespread disease commonly associated with dentin hypersensitivity, that, in turn, may complicate routine dental care, leading to plaque accumulation. We aimed to assess the antigingivitis, desensitizing, and antiplaque effects of a fluoride-containing (TWF) alkaline toothpaste and a fluoride-free (TW) alkaline toothpaste. Eighty-four consenting patients aged 20–25 years with diagnosed gingivitis and dentin hypersensitivity (DH) were recruited in this double-blind, parallel-group study and randomly divided into two groups (each *n* = 42). Eighty-two patients completed the entire study protocol. The outcomes were assessed after 4 weeks of intervention. A significant improvement in gingival condition was found according to the modified gingival index, with effect sizes of 0.99 [CI95%: 0.52–1.46] and 1.71 [CI95%: 1.18–2.24], and the gingival bleeding index, with effect sizes of 3.17 [CI95%: 2.39–3.94] and 2.64 [CI95%: 1.96–3.32] in the TW and TWF groups, respectively. DH also decreased in both groups, with a significantly greater reduction in the TWF group (effect sizes of 3.28 [CI95%: 2.51–4.04] and 3.10 [CI95%: 2.40–3.80] according to the visual analog scale and Schiff scale, respectively). No side effects were registered. In conclusion, the use of alkaline toothpaste provided a significant reduction in gingival inflammation and bleeding, DH, and oral hygiene after 4 weeks of daily use in young adults. Trial Registration: NCT0562376. Funding: none.

## 1. Introduction

Gingivitis is defined as “an inflammatory lesion resulting from interactions between the dental plaque biofilm and the host’s immune-inflammatory response, which remains contained within the gingiva and does not extend to the periodontal attachment” and affects more than 90% of the world’s population [1]. Biofilm control is crucial for the prevention of periodontal diseases [2]. Plaque should be maintained at levels compatible with health in order to preserve the host microbial dynamic balance, which decreases the risk of disease [3].

However, bacteria are a necessary but not sufficient requirement for the development of periodontal diseases, and host-related factors should also be considered [4,5]. Apart from other oral environmental conditions, i.e., nutrition, atmosphere, and redox potential [6], dental biofilm growth rate may be influenced by temporary alterations of oral acidity or alkalinity [6,7]. The mean pH of saliva is 6.7, within the normal range of 6.2–7.6 [8]. The resting oral pH may vary between 5.0 and 9.0 depending on different factors, including age [9], gender [9,10], season, and even time of day [10]. Salivary pH alterations have been found in patients with various diseases, such as anorexia nervosa [11], gastroesophageal reflux disease [12,13,14], and diabetes mellitus [15,16]. Hormonal changes have also been shown to influence this parameter [17,18,19,20,21]. 

A saliva pH of around 7.0 is usually associated with a healthy state of teeth and periodontium. In this environment, there is a low risk of dental caries and calculus formation [22]. At neutral pH, saliva inhibits bacterial glycolysis, thus preventing the development of anaerobic conditions [7]. Values below 7.0 indicate acidity; at this pH, the oral cavity is more susceptible to dental caries, periodontal diseases, and halitosis [8,22]. A study by Takashi et al. showed that the growth of periodontal pathogens (*P. intermedia*, *F. nucleatum*, and *P. gingivalis*) occurs mainly at a pH of 5.0–7.0 [23,24]. 

Furthermore, low oral pH values may cause dental erosion (dissolution of hard tooth tissues by acidic substances) [25]. Apart from the loss of tooth structure, eroded dentin is commonly characterized by the opening and enlargement of dentinal tubules, increased permeability, and consequently, dentinal hypersensitivity (DH) [26,27]. Acidic conditions in the oral cavity and the resulting erosive tooth wear have been associated with DH, especially in young patients [28]. In turn, hypersensitive cervical lesions may make brushing and routine dental care uncomfortable, thus leading to further plaque accumulation and deterioration of gingival health [29]. In a study by Taani et al., about 11% of patients with DH avoided normal oral hygiene and had poor eating habits [30].

Based on the etiology and mechanisms leading to DH, its conventional management aims to occlude dentinal tubules or create precipitates inside them [31,32,33]. Desensitizing treatment may include the use of physical [34] or chemical methods. Chemical agents most commonly include various fluorinated derivatives, calcium apatites, and bioactive glass [35,36,37], and are prescribed in the form of over-the-counter toothpaste [38]. Frequent application of fluoride-containing products has been specifically recommended to prevent further progression of erosion and hard tooth tissue demineralization [36,39,40].

However, there are controversial findings on the correlation between the remineralizing potential of various fluoride-containing agents and the pH of the environment [41,42]. It can be hypothesized that pH control may be effective in patients with gingivitis and DH by affecting both periodontal pathogens and hard tooth tissue mineralization. A greater mineralization potential has been found in patients with increased pH [43,44]. An alkaline pH is associated with increased proteolytic activity and promotes calcium phosphate deposition [45]; thus, an alkaline pH may potentially provide enhanced remineralization of hard tooth tissues and plugging of the dentinal tubules. To the best of our knowledge, few clinical trials have investigated the combined anti-inflammatory and desensitizing effects of toothpaste [46,47]. Habashneh et al. concluded that a triclosan/copolymer/fluoride toothpaste possessed an antigingivitis effect, but did not influence DH [46]. Monterubbianesi et al. reported a combined desensitizing and anti-inflammatory effect of a calcium sodium phosphosilicate bioactive glass toothpaste [47]. Moreover, with the development of new oral care products, it is necessary to continuously update the evaluations of their effects.

The aim of this study was to compare the effect of a toothpaste containing alkaline thermal water with fluoride and a toothpaste containing alkaline thermal water without fluorinated derivatives, on gingivitis, dentin hypersensitivity, and oral hygiene in young adults. The tested null hypothesis was that there would be no differences in the antigingivitis, desensitizing, and antiplaque effects of the fluoride-free and fluoride-containing alkaline toothpastes.

## 2. Materials and Methods

### 2.1. Ethical Approval

This study was approved by the Ethics Committee of Sechenov University (Protocol No. 06-22) and registered on ClinicalTrials.gov (No. NCT05623761).

### 2.2. Study Design

This double-blind, randomized, two-arm parallel-group study was conducted from November 2022 to January 2023 at Sechenov University (Moscow, Russia).

### 2.3. Sampling Criteria

Eighty-four adults of both genders aged 20–25 years with clinically diagnosed DH and gingivitis [48] were enrolled and randomized by two study authors with more than 5 years of clinical experience each (MP and EA). Participants provided their written informed consent for participation in the study and publication of the data for research and education purposes.

The sample size was determined for a two-tailed Wilcoxon matched-pairs signed-rank test with the assumption that the effect size would be medium (0.5). Sample size calculations were performed using G*Power (version 3.1.9.6) free software: the alfa-level was set as 0.05 and the power was set at 80% (allocation ratio = 1). The target sample size comprised 42 participants in each group (39 participants according to sample size calculations plus 7% to account for possible dropout), giving 84 patients in total.

#### 2.3.1. Inclusion Criteria

- Age 20–25 years;
- Signed an approved Informed Consent Form, authorizing the participation in the trial and use of the results of the trial for education purposes and publication;
- Clinically diagnosed gingivitis;
- At least one tooth with clinically diagnosed DH.

#### 2.3.2. Exclusion Criteria

- Medical and pharmacotherapeutic histories that may compromise the protocol (pregnancy or breastfeeding, psychiatric disorders, allergies to toothpaste ingredients, eating disorders, etc.);
- Systemic conditions that are etiologic to DH (e.g., chronic acid regurgitation);
- History of chemotherapy or radiotherapy;
- Antibiotic, anti-inflammatory, or anti-coagulant therapy 4 weeks prior to or after the baseline visit;
- An oral mucosa pathology;
- Periodontal surgery within the previous 3 months;
- Orthodontic treatment within the previous 3 months;
- Any other pathology or teeth defects accompanied by pain;
- Use of any other agents for DH management 4 weeks prior to or after the baseline visit;
- Teeth with large restorations and/or teeth with restorations in the cervical area;
- Dental bleaching within the previous 3 months;
- Withdrawal of consent;
- Noncompliance with the study procedures;
- An adverse event that required treatment discontinuation.

### 2.4. Randomization

Subjects meeting all the inclusion and none of the exclusion criteria were randomized at the baseline visit into the TWF (thermal water and fluoride toothpaste) or TW (thermal water toothpaste) groups (1:1 ratio) in accordance with a computer-generated schedule prepared by a third-party (Table 1). The allocation concealment was ensured by using sealed containers. Toothpastes in white bottles without any titles were placed in the containers and numbered by a person who did not participate in the study. Each patient received a sealed container with a toothpaste on enrolment. Neither the patients nor researchers were aware of the type of intervention.

### 2.5. Interventions

At baseline, study participants received identical soft toothbrushes, and oral hygiene instructions were provided. The modified Bass technique was taught by two study operators (MP and EA). During the study, the patients brushed their teeth two times a day. Control examinations were performed at baseline and after 4 weeks. All clinical examinations were performed by two researchers (MP and EA). The examiners had been trained and calibrated to obtain an intra- and inter-examiner agreement of 92%, using Kappa statistics. During the first visit, demographic data, medical history, and medication history were registered. 

All patients were assessed using the following indices and scales: Modified Gingival Index (MGI), Gingival Bleeding Index (BI), Visual Analogue Scale (VAS), Schiff Scale (SS), and Rustogi Modified Navy Plaque Index (RMNPI). Their salivary pH levels were measured, and adverse events were recorded.

Participants were free to withdraw from the study at any point.

### 2.6. Outcomes

Primary outcome measures included changes in MGI, BI, and DH scores according to the SS and VAS scales. Secondary outcome measures included changes in salivary pH and RMNPI.

#### 2.6.1. MGI

The MGI was used to assess visual symptoms of gingival inflammation. Two scores were recorded buccally/labially, and two were recorded lingually/palatally (whole mouth). The following scores were assigned:

0—Absence of inflammation;
1—Mild inflammation; slight change in color; little change in texture of any portion of the marginal or papillary gingival unit;
2—Mild inflammation; criteria as above plus the entire marginal or papillar gingival unit;
3—Moderate inflammation; glazing, redness, edema, and/or hypertrophy of the marginal or papillary gingival unit;
4—Severe inflammation; marked redness, edema and/or hypertrophy of the marginal or papillary gingival unit, spontaneous bleeding, congestion, or ulceration.

#### 2.6.2. BI

The BI was used to assess gingival bleeding on probing. After air-drying, bleeding caused by gentle probing of the gingival crevice was assessed by the investigator. A probe was inserted to a depth of 1 mm and moved around the tooth. Three scores were recorded on each facial and lingual gingival surface on a whole-mouth basis. The following scores were assigned:

0—No bleeding after 30 s;
1—Bleeding upon probing after 30 s;
2—Immediate bleeding.

#### 2.6.3. Sensitivity Testing

To assess DH, several approaches were used. First, the VAS was used to score subjective pain intensity in response to evaporative stimuli. Second, the SS was used to evaluate DH objectively. In each patient, maximum SS values and mean SS values across all sensitive teeth were analyzed. 

The tooth was isolated, and a blast of air from an air-water syringe (60 ± 5 psi, 18–22 °C) was directed onto the middle third of the buccal surface for 1 s from a distance of approximately 10 mm. Subjective sensitivity was reported by the patients using the VAS (10-point scale). A score of 0 indicated no pain; a score of 10 indicated intense pain.

Teeth sensitivity was also assessed by the operator in accordance with the Schiff criteria:

0—No reaction;
1—Discomfort, but the patient does not insist on stopping the test;
2—Discomfort accompanied by a request to discontinue the test;
3—Severe pain and pronounced motor response, which meant that the test was immediately discontinued.

#### 2.6.4. Salivary pH

Unstimulated whole saliva samples were collected from 10 a.m. to 11 a.m. Study participants refrained from eating, drinking, smoking, or conducting oral hygiene for a minimum of 90 min prior to the procedure. The participants were comfortably seated, avoiding swallowing saliva, and spat all the saliva they produced into a calibrated tube until the required volume was collected. The pH was measured immediately after the collection using a digital pH meter (MILWAUKEE PH56 PRO, Rocky Mount, NC, USA). Then, the participants were asked to brush their teeth with the assigned toothpaste, and pH measurements were repeated one minute after toothbrushing.

#### 2.6.5. RMNPI

Nine zones per each facial and lingual tooth surface were examined; a maximum of 504 sites (excluding 3rd molars, and teeth with artificial crowns or cervical restorations). Disclosed plaque was scored in each tooth area as follows: 0—absent, and 1—present. A mean plaque index was determined for each patient on a whole-mouth basis [49]. 

#### 2.6.6. Toothpaste pH

To prepare a solution for toothpaste pH measurement, the toothpastes were mixed with deionized water (ratio 1:3) [50], and a pH meter (MILWAUKEE PH56 PRO, Rocky Mount, NC, USA) was used to assess pH values in each slurry. The measurements were repeated 5 times for each toothpaste; mean pH values and standard deviations are presented in Table 1.

### 2.7. Statistical Analysis

A Kolmogorov–Smirnov test was used to assess the normality of the distribution. A Wilcoxon matched-pairs signed-rank test was performed to compare quantitative variables in the dependent groups and a Mann–Whitney U test was performed to compare quantitative variables in the independent groups. The proportions of Schiff scores were compared using Fisher’s exact test (between the TW and TWF groups at each time point) and McNemar’s test (within the study groups). Cohen’s d was used to assess the effect size in each group by comparing mean values at baseline and 4 weeks.

### 2.8. Data Management

Data were entered in the MS Excel database and then exported into the CSV file format and analyzed in R, version 3.6.0 (26 April 2019) with the following packages: “doBy”, “rstatix”, “stats”, and “effectsize” in RStudio, version 1.2.1335 2009-2019. All patients who did not substantially deviate from the protocol (which was determined on a per-subject basis by the study’s principal investigator) were analyzed immediately before the database lock.

## 3. Results

### 3.1. Patient Flow and Demographics

**Figure 1:**
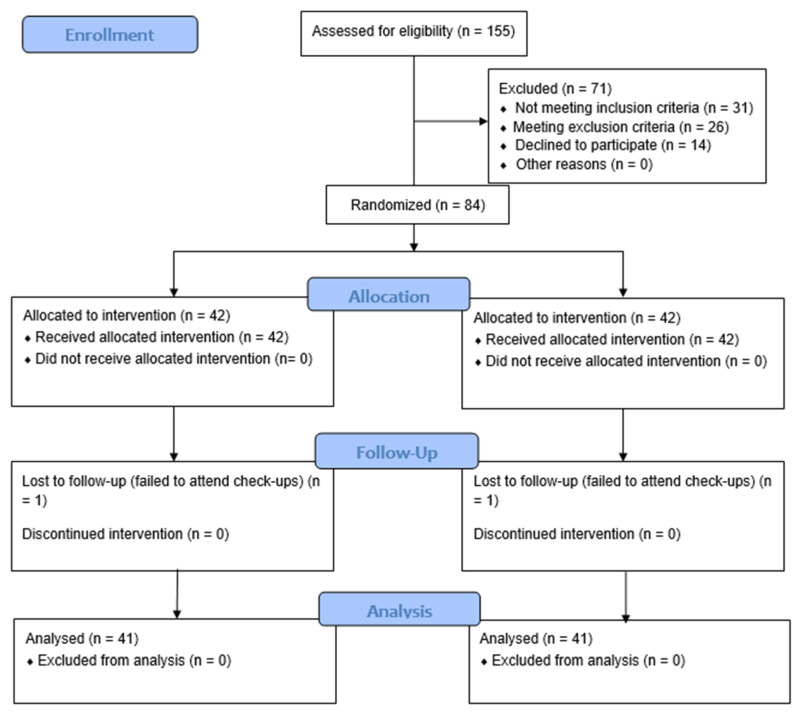
Participant flow diagram.

One hundred and fifty-five volunteers aged 20–25 years were assessed for eligibility to be enrolled in the study. Thirty-one patients did not meet the inclusion criteria (21 patients had no teeth, with DH confirmed clinically, 8 patients had no gingivitis, and 2 patients refused to sign the informed consent form). Twenty-six patients met at least one of the exclusion criteria (18 patients had systemic conditions etiologic to DH, 4 patients underwent dental bleaching within the previous 4 months, 2 patients used desensitizing toothpaste, and 2 had taken antibiotics). Eighty-four patients were enrolled and randomly assigned to the following study groups: the TW group (*n* = 42) and the TWF group (*n* = 42). Two patients (one from each group) were lost to follow-up. Eighty-two patients were included in the final analysis (Figure 1 and Table 2). No patients reported adverse effects.

### 3.2. Gingival Condition Assessment

Gingival condition was assessed using MGI and BI. The MGI values ranged from 0.006 to 0.143 points at baseline, and no significant differences were found between the groups (*p* = 0.9113). At 4 weeks, a significant improvement in gingival condition was observed in the TW and TWF groups (*p* < 0.001), and the mean MGI values were 0.030 and 0.017 points, respectively (*p* < 0.001) (Table 3).

The baseline BI values ranged from 0.05 to 0.40 points. The Mann–Whitney test revealed no significant differences between the groups both at baseline (*p* = 0.3557) and after 4 weeks (*p* = 0.8303). At 4 weeks, a significant reduction in gingival bleeding was registered in both groups (*p* < 0.001) (Table 4). 

### 3.3. Dentin Hypersensitivity Assessment

The VAS was used to score subjective pain intensity. According to VAS sensitivity testing, there were no significant differences between the study groups at baseline (*p* = 0.3231). At 4 weeks, subjective pain intensity decreased significantly in the TW and TWF groups (*p* = 0.00714 and *p* < 0.001, respectively); however, the TWF group demonstrated significantly lower values (Table 5). 

The SS was used to evaluate DH objectively. In each patient, maximum SS values across all sensitive teeth were analyzed (Table 6 and Table 7).

The maximum SS values did not differ significantly between the TW and TWF groups (*p* = 0.3692). At 4 weeks, a significant decline in this parameter was found in the TW and TWF groups (*p* < 0.001). The decrease was 1.0 and 1.9 points, respectively (*p* < 0.001), with a more pronounced reduction in the TWF group (*p* < 0.001). At 4 weeks, the SS scores in both groups were significantly lower than at baseline (Table 6). 

Table 7 shows the distribution of maximum SS values across the study groups at different time points.

### 3.4. Oral Hygiene Assessment

The baseline oral hygiene levels (according to RMNPI) in the study groups were similar (*p* = 0.4637). We observed a significant improvement at 4 weeks in both groups (*p* < 0.001), without significant differences between them (*p* = 0.9592) (Table 8). 

### 3.5. Salivary pH Assessment

The mean salivary pH value in patients with gingivitis (whole study sample) was 7.04 ± 0.34. Before brushing, there were no significant differences in the mean pH levels between the TW and TWF groups (7.07 ± 0.32 and 6.99 ± 0.39, respectively). After 2 min of toothbrushing, a significant increase in this parameter was observed in the TW and TWF groups, which was 0.29 (*p* = 0.02275) and 0.51 (*p* = 0.006558) points, respectively (*p* = 0.272) (Table 9).

## 4. Discussion

The study compared the effect of alkaline (fluoride-containing and fluoride-free) toothpastes on oral health indicators. We found a significant improvement in gingival condition and oral hygiene in both groups; dentin hypersensitivity also decreased in both groups, with a significantly greater decrease in the TWF group. Therefore, the tested null hypothesis was accepted for antigingivitis and antiplaque effects; however, it was rejected for the desensitizing effect. The salivary pH became more alkaline after the use of both toothpastes. 

The use of the tested toothpastes had a positive impact on gingival health. At 4 weeks, a significant reduction in the symptoms of gingivitis was observed in the TW and TWF groups according to MGI (the effect sizes were 0.99 and 1.71, respectively) and BI (the effect sizes were 3.17 and 2.64, respectively). As gingivitis is a biofilm-mediated condition, this may be explained by an improvement in oral hygiene. We found that the levels of oral hygiene increased significantly in both groups (the effect sizes were 2.76 and 2.96 for the TW and TWF groups, respectively). Cleaning effectiveness depended on the characteristics of a toothpaste, mainly abrasivity, as measured by the relative dental abrasivity (RDA) value [51,52] and environmental factors, including pH [53,54]. As high abrasivity may lead to dentin wear and DH [53], in patients with DH, toothpastes with low RDA are recommended [55]. Low-abrasive toothpastes are most commonly characterized by an RDA of less than 70 [56,57,58]. As the RDA values of the toothpastes tested in this study were low (7 and 17 in the TW and TWF groups, respectively), a significant improvement in oral hygiene in both groups was possibly due to some other factors, e.g., the alkalinity of the toothpaste. It is claimed by the manufacturer that the TW and TWF toothpastes contain 46% of alkaline thermal water (Castéra-Verduzan, pH = 8.1). We found the pH values of the tested toothpastes themselves were 8.8 ± 0.2 and 8.8 ± 0.1 for TW and TWF, respectively. Besides, the observed improvement in oral hygiene may be explained by the Hawthorne effect [46,59].

Apart from the effect of the studied toothpastes on oral hygiene and gingival health, we also assessed their influence on DH. The first line of DH management may include the use of an over-the-counter toothpaste containing fluoride compounds [60,61]. In the present study, the TWF toothpaste contained sodium fluoride (1450 ppm), while the TW toothpaste was fluoride-free. Maximum SS and VAS values were significantly lower in the TWF group. Apart from the F concentration, some other factors may have influenced the clinical effect of the fluoride-containing toothpaste, e.g., frequency and application time, and local pH [41]. An in vitro study by Lammers et al. showed that a higher mineral content was found in bovine enamel after the application of 0.03 ppm fluoride at pH 6.8 than at pH 5.5 [41]. Yamazaki et al. reported that neutral solution provided preferential remineralization of the outer portion of the surface-softened lesion [42], which is of particular importance to minimize the effect of erosion and reduce DH [26]. In a study by Lussi et al., an alkaline toothpaste with pH 9.37 showed slightly better rehardening of the acid-softened enamel surface [62]. 

Surprisingly, a slight yet significant decrease in DH was registered in the TW group. The desensitizing effect of fluoride-free toothpaste could also have been associated with enhanced mineralization of the tooth surfaces in non-acidic environments. Additionally, improved oral hygiene could have contributed to a decrease in DH in both groups, as a positive correlation had been demonstrated between plaque and dentin hypersensitivity [63].

To assess the effect of the alkaline toothpaste on salivary pH, we measured its values before and after toothbrushing. There have been controversial findings on salivary pH in patients with periodontal diseases in the literature [8,22,64,65]. In the present study, we enrolled patients with clinically diagnosed gingivitis. Initially, there were no significant differences in the mean pH levels in the TW and TWF groups (7.07 ± 0.32 and 6.99 ± 0.39, respectively). These results in subjects with gingivitis are in agreement with the findings of Orosco et al. In their study, the mean initial salivary pH in the gingivitis group was 7.01 ± 0.25 [66]. In the studies by Garcia et al. [67] and Koppolu et al. [8], salivary pH levels in patients with gingivitis were slightly higher (7.3 and 7.21 ± 0.11, respectively). In this study, after 2-min toothbrushing, a significant increase in salivary pH was found in the TWF group. The mean pH value was 7.50 ± 0.23, and the effect size was 1.59. An increase in salivary pH has also been reported in other studies assessing the effect of brushing the teeth with fluoride-containing toothpaste. A study by Setiawan et al. reported a significant increase in salivary pH after 2-min toothbrushing with fluorinated toothpaste (from 7.174 ± 0.253 to 7.595 ± 0.229) [68]. Fibryanto et al. found that salivary pH increased from 7.22 ± 0.16 to 7.42 ± 0.13 after brushing with sodium monofluorophosphate toothpaste [69]. In this study, the mean difference between the values before and after brushing in the TWF group was greater than the mean difference between the values reported in the aforementioned studies. Besides, we also observed a significant rise in pH values in the TW group (the mean pH value was 7.36 ± 0.33, effect size 0.89). It can be hypothesized that changes in pH values may be explained not only by the effect of fluoride but also by the effects of toothbrushing itself (e.g., increase in salivation) and toothpaste alkalinity.

We readily acknowledge several limitations to our study. First, both toothpastes were alkaline and no comparison was made with acidic or neutral formulations. Next, we enrolled patients of a limited age group (20–25-year-olds). It is possible that in older age groups the effects of the tested toothpastes will be different from ours due to changes in dentin mineralization and structure. Although at baseline there were no significant differences between the groups, a greater number of females in both groups may have impacted the results. In addition, a longer follow-up is required to assess the effect of prolonged use of the assessed toothpastes. Further studies should include patients from populations of different regions and ages and have a longer follow-up period.

## 5. Conclusions

According to our findings, the toothpaste with alkaline thermal water provided a significant reduction in gingival inflammation and bleeding, dental plaque accumulation, and dentin hypersensitivity after 4 weeks of daily use in young adults. The effect on DH was significantly greater for the fluoride-containing toothpaste (1450 ppm) than for the fluoride-free toothpaste. However, more research with broader age groups and longer follow-up periods is warranted to evaluate the long-term effect of this formulation.

## Figures and Tables

**Table 1:** The characteristics of the tested toothpastes.

Group	Toothpaste Composition	Active Ingredient Description/RDA/pH
TW	Aqua (Castéra-Verduzan Thermal Spring water), Glycerin, Hydrogenated Starch Hydrolysate, Aqua, Hydrated Silica, Decyl Glucoside, Cellulose Gum Aroma, Sodium Benzoate, Stevia Rebaudiana Extract, Sodium Hydroxide, Limonene.	Castéra-Verduzan Thermal Spring water RDA 7 pH = 8.8 ± 0.2
TWF	Aqua (Castéra-Verduzan Thermal Spring water), Glycerin, Hydrogenated Starch Hydrolysate, Aqua, Hydrated Silica, Decyl Glucoside, Cellulose Gum Aroma, Sodium Benzoate, Sodium Fluoride, Stevia Rebaudiana Extract, Sodium Hydroxide, Limonene, Sodium Fluoride: 1450 ppm.	Castéra-Verduzan Thermal Spring water, Sodium Fluoride: 1450 ppm RDA 17 pH = 8.8 ± 0.1

TW—toothpaste containing thermal water; TWF—toothpaste containing thermal water with fluoride; RDA—relative dentin abrasivity.

**Table 2:** Subject demographics.

Tested Toothpaste	TW	TWF	*p* Value
Sex n (%)			
Female	30 (73.2)	34 (82.9)	0.4241 ^1^
Male	11 (26.8)	7 (17.07)	
Total	41 (100)	41 (100)	
Age			
Mean (sd)	21.39 (1.53)	21.05 (1.34)	0.2498 ^2^
Median (Q1, Q3)	21 (20, 22)	21 (20, 21)	
Min, Max	20, 25	20, 25	

^1^ Fisher’s exact test; ^2^ Wilcoxon signed-rank test.

**Table 3:** MGI values.

Group	Baseline	4 Weeks	Effect Size (Cohen’s d)
TW (*n* = 41)			
Mean (SD)	0.053 (0.029) ^a^	0.030 (0.016) ^A^	0.99
CI95%	0.044–0.062	0.025–0.035	0.52–1.46
Median (Q1, Q3)	2.86 (1.93; 3.43)	0.63 (0.43; 0.78)	
*p* values	*p* < 0.001 ^1^		
TWF (*n* = 41)			
Mean (SD)	0.053 (0.024) ^a^	0.017 (0.013) ^B^	1.71
CI95%	0.043–0.058	0.013–0.021	1.18–2.24
Median (Q1, Q3)	2.46 (1.96; 3.21)	0.54 (0.43; 0.86)	
*p* values	*p* < 0.001 ^1^		

^a, A, B^ Different letters indicate statistically significant differences between the groups; ^1^ Wilcoxon signed-rank test; MGI—Modified Gingival Index; TW—toothpaste containing thermal water; TWF —toothpaste containing thermal water with fluoride.

**Table 4:** BI values.

Group	Baseline	4 Weeks	Effect Size (Cohen’s d)
TW (*n* = 41)			
Mean (SD)	0.21 (0.07) ^a^	0.05 (0.02) ^A^	3.17
CI95%	0.19–0.23	0.05–0.06	2.39–3.94
Median (Q1, Q3)	0.22 (0.17; 0.26)	0.05 (0.04; 0.07)	
*p* values	*p* < 0.001 ^1^		
TWF (*n* = 41)			
Mean (SD)	0.20 (0.07) ^a^	0.05 (0.02) ^A^	2.64
CI95%	0.18–0.22	0.05–0.06	1.96–3.32
Median (Q1, Q3)	0.20 (0.14; 0.25)	0.05 (0.04; 0.07)	
*p* values	*p* < 0.001 ^1^		

^a, A^ Different letters indicate statistically significant differences between the groups; ^1^ Wilcoxon signed-rank test; BI—gingival Bleeding Index; TW—toothpaste containing thermal water; TWF—toothpaste containing thermal water with fluoride.

**Table 5:** VAS sensitivity values.

Group	Baseline	4 Weeks	Effect Size (Cohen’s d)
TW (*n* = 41)			
Mean (SD)	6.8 (2.5) ^a^	6.2 (2.6) ^A^	0.23
CI95%	6.0–7.5	5.4–7.0	−0.21–0.66
Median (Q1, Q3)	7 (5; 9)	7 (3; 8)	
*p* values	*p* = 0.00714 ^1^		
TWF (*n* = 41)			
Mean (SD)	6.3 (2.2) ^a^	0.8 (0.9) ^B^	3.28
CI95%	5.6–7.0	0.5–1.0	2.51–4.04
Median (Q1, Q3)	6 (5; 8)	1 (0; 1)	
*p* values	*p* < 0.001 ^1^		

^a, A, B^ Different letters indicate statistically significant differences between the groups; ^1^ Wilcoxon signed-rank test; VAS—Visual Analogue Scale; TW—toothpaste containing thermal water; TWF—toothpaste containing thermal water with fluoride.

**Table 6:** Maximum SS values.

Group	Baseline	4 Weeks	Effect Size (Cohen’s d)
TW (*n* = 41)			
Mean (SD)	2.2 (0.8) ^a^	1.2 (0.7) ^A^	1.30
CI95%	2.0–2.5	1.0–1.5	0.82–1.78
Median (Q1, Q3)	2 (2; 3)	1 (1; 2)	
*p* values	*p* < 0.001 ^1^		
TWF (*n* = 41)			
Mean (SD)	2.1 (0.8) ^a^	0.2 (0.4) ^B^	3.10
CI95%	1.9–2.3	0.1–0.3	2.40–3.80
Median (Q1, Q3)	2 (2; 3)	0 (0; 0)	
*p* values	*p* < 0.001 ^1^		

^a, A, B^ Different letters indicate statistically significant differences between the groups; ^1^ Wilcoxon signed-rank test; SS—Schiff scale; TW—toothpaste containing thermal water; TWF—toothpaste containing thermal water with fluoride.

**Table 7:** Distribution of the maximum SS scores across the study groups, abs. (%).

Score	TW (0 w) ^1^	TW (4 w) ^2^	TWF (0 w)	TWF (4 w)
0	-	-	-	33 (80)
1	9 (22)	7 (17)	10 (24)	8 (20)
2	13 (32)	17 (41.5)	17 (41)	-
3	19 (46)	17 (41.5)	14 (34)	-
	*p* < 0.001 ^3^		*p* < 0.001 ^3^	

^1^ Between-group *p*-value = 0.5605 (Fisher’s exact test) at baseline; ^2^ Between-group *p*-value < 0.001 (Fisher’s exact test) at 4 weeks; ^3^ Within-group *p*-values (McNemar’s chi-square test); SS—Schiff scale; TW—toothpaste containing thermal water; TWF—toothpaste containing thermal water with fluoride.

**Table 8:** RMNPI index values.

Group	Baseline	4 Weeks	Effect Size (Cohen’s d)
TW (*n* = 41)			
Mean (SD)	2.85 (1.04) ^a^	0.62 (0.25) ^A^	2.76
CI95%	2.53–3.17	0.55–0.70	2.03–3.48
Median (Q1, Q3)	2.86 (1.93; 3.43)	0.63 (0.43; 0.78)	
*p* values	*p* < 0.001 ^1^		
TWF (*n* = 41)			
Mean (SD)	2.73 (1.07) ^a^	0.61 (0.21) ^A^	2.96
CI95%	2.41–3.06	0.55–0.68	2.20–3.70
Median (Q1, Q3)	2.46 (1.96; 3.21)	0.54 (0.43; 0.86)	
*p* values	*p* < 0.001 ^1^		

^a, A^ Different letters indicate statistically significant differences between the groups; ^1^ Wilcoxon signed-rank test; RMNPI—Rustogi Modified Navy Plaque Index; TW—toothpaste containing thermal water; TWF—toothpaste containing thermal water with fluoride.

**Table 9:** Salivary pH values before and after toothbrushing.

Group	Before	After	Effect Size (Cohen’s d)
TW (*n* = 41)			
Mean (SD)	7.07 (0.32) ^a^	7.36 (0.33) ^A^	0.89
CI95%	6.88–7.27	7.16–7.55	0.13–1.64
Median (Q1, Q3)	7.10 (7.00; 7.20)	7.40 (7.05; 7.55)	
*p* values	*p* = 0.02275 ^1^		
TWF (*n* = 41)			
Mean (SD)	6.99 (0.39) ^a^	7.50 (0.23) ^A^	1.59
CI95%	6.87–7.11	7.43–7.57	0.58–2.57
Median (Q1, Q3)	7.00 (6.80; 7.25)	7.60 (7.30; 7.65)	
*p* values	*p* = 0.006558 ^1^		

^a, A^ Different letters indicate statistically significant differences between the groups; ^1^ Wilcoxon signed-rank test; TW—toothpaste containing thermal water; TWF—toothpaste containing thermal water with fluoride.

## Data Availability

The datasets used and/or analyzed during the current study are available from the corresponding author upon reasonable request.
